# Mechanism-based inhibition of an aldolase at high concentrations of its natural substrate acetaldehyde: structural insights and protective strategies†

**DOI:** 10.1039/c5sc04574f

**Published:** 2016-03-30

**Authors:** Markus Dick, Rudolf Hartmann, Oliver H. Weiergräber, Carolin Bisterfeld, Thomas Classen, Melanie Schwarten, Philipp Neudecker, Dieter Willbold, Jörg Pietruszka

**Affiliations:** a Institute of Bioorganic Chemistry, Heinrich-Heine-Universität Düsseldorf im Forschungszentrum Jülich 52426 Jülich Germany j.pietruszka@fz-juelich.de; b Institute of Complex Systems, ICS-6: Structural Biochemistry, Forschungszentrum Jülich GmbH 52425 Jülich Germany; c Institute of Bio- and Geosciences, IBG-1: Biotechnology, Forschungszentrum Jülich GmbH 52425 Jülich Germany; d Institut für Physikalische Biologie, Heinrich-Heine-Universität Düsseldorf 40225 Düsseldorf Germany

## Abstract

2-Deoxy-d-ribose-5-phosphate aldolase (DERA) is used in organic synthesis for the enantioselective reaction between acetaldehyde and a broad range of other aldehydes as acceptor molecules. Nevertheless, its application is hampered by a poor tolerance towards high concentrations of acetaldehyde, its natural substrate. While numerous studies have been performed searching for new, more acetaldehyde-resistant DERAs, the mechanism underlying this deactivation process has remained elusive. By using NMR spectroscopy on both the protein and the small-molecule scale, we could show that a reaction product binds to the inner part of the enzyme, and that this effect can be partly reversed *via* heating. The crystal structure of DERA before and after acetaldehyde incubation was determined at high resolution, revealing a covalently bound reaction product bridging the catalytically active lysine (K167) to a nearby cysteine (C47) in the deactivated enzyme. A reaction mechanism is proposed where crotonaldehyde as the aldol product of two acetaldehyde molecules after water elimination forms a Schiff base with the lysine side chain, followed by Michael addition of the cysteine thiol group to the C_β_ atom of the inhibitor. In support of this mechanism, direct incubation of DERA with crotonaldehyde results in a more than 100-fold stronger inhibition, compared to acetaldehyde, whereas mutation of C47 gives rise to a fully acetaldehyde-resistant DERA. Thus this variant appears perfectly suited for synthetic applications. A similar diagnostic and preventive strategy should be applicable to other biocatalysts suffering from mechanism-based inhibition by a reactive substrate, a condition that may be more common than currently appreciated in biotechnology.

## Abstract 2

Understanding the deactivation mechanism of 2-deoxy-d-ribose-5-phosphate aldolase by its natural substrate leads to a single mutant showing complete acetaldehyde resistance.

## Graphical abstract

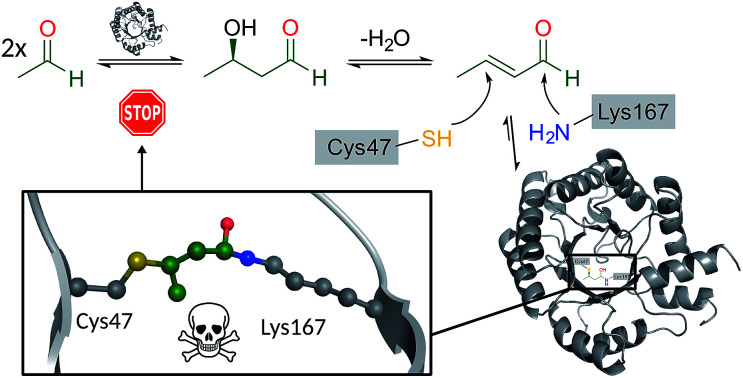

## Introduction

The formation of C–C-bonds *via* an asymmetric aldol reaction between two carbonyl compounds is a commonly utilized reaction in organic synthesis.^1^ 2-Deoxy-d-ribose-5-phosphate aldolase (DERA) catalyzes the reaction in a highly stereo-selective manner and thus has become an important tool to synthesize chiral building blocks for natural products.^2–4^ In 2001 the catalytic mechanism was resolved for DERA from *Escherichia coli* (DERA_EC_) by Heine *et al.*: the active K167 forms a Schiff base with the donor molecule, while K201 functions to reduce the p*K*_a_ of K167 and, together with D102, participates in a proton-relay system critical for efficient catalysis.^5^ As acceptor molecule a broad range of aldehydes and ketones can be used. In contrast, the donor substrate is limited to acetaldehyde.^2,6^ Hence, DERA is described as an acetaldehyde-dependent aldolase.

**Fig. 1:**
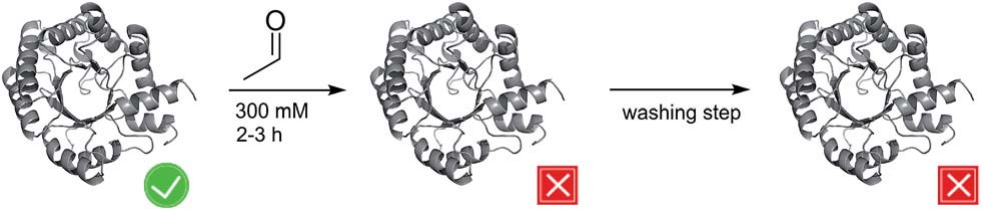
Schematic representation of the effect of acetaldehyde on DERA activity. The active and inactive states are labeled by a tick and a cross, respectively.

However, practical application of DERA in organic synthesis is limited by its poor stability towards high acetaldehyde concentrations.^7,8^ After incubation with 200 to 300 mM acetaldehyde, the enzyme is irreversibly inactivated, and activity cannot be restored upon washing with acetaldehyde-free buffer (Fig. 1). In the past decades, significant efforts have been made to overcome this bottleneck. Several new DERAs from different organisms have been characterized showing a higher acetaldehyde tolerance.^9–16^ Most promising examples were found in thermophilic organisms (up to 70% remaining activity after overnight incubation with 300 mM acetaldehyde), suggesting a correlation between acetaldehyde and thermal stability.^11,17^ Notably, a lack of activity at ambient temperatures (which are used in DERA-catalyzed reactions) has hampered their adoption in commercial applications. All of these new aldolases have in common that they are usually expressed heterologously in *E. coli*, implying lower protein yields compared to *E. coli* DERA, which is over-expressed in its cognate host. In this study we focus on two main questions: (1) by which mechanism does acetaldehyde deactivate the enzyme? (2) Is there a way to prevent/withdraw this effect? Until now there has only been one study by Jennewein *et al.*^8^ where efforts were made to understand the mechanisms underlying this deactivation process. They proposed Schiff base formation between acetaldehyde and exposed lyzine residues leading to inactivation of the enzyme. Notably, single point mutations of these amino acids did not improve stability. Here we report that acetaldehyde indeed forms Schiff bases with most (outer) lysines, but that this reaction is not responsible for deactivation. Rather, we have identified a reaction product that blocks the catalytic center by covalently linking the active lysine to a nearby cysteine residue. Finally, we have developed two different strategies to counteract deactivation of DERA by this mechanism-based inhibitor.

## Results and discussion

### Analysis of Schiff base formation

Our initial hypothesis was based on the idea that acetaldehyde reacts with outer lysines of DERA, forming Schiff bases. This effect would increase the hydrophobicity of the outer protein shell due to a reduced p*K*_a_ of the lysine sidechain – resulting in an uncharged residue at neutral pH^18^ – and might cause partial unfolding of the protein due to the decrease of the polarity gradient between the hydrophobic core and the peripheral regions. Analysis *via* mass spectrometry after incubation of DERA with acetaldehyde and a subsequent reducing step with sodium borohydride revealed an ethylation pattern in 14 of 16 outer lysines. Thus it could be indirectly shown that acetaldehyde forms Schiff bases with the lysines on the protein surface. To find out whether there is a relationship between the number of outer lysines and the stability of DERA towards high acetaldehyde concentrations, the genome database was searched for DERA genes coding for a protein with fewer lysine residues. The orthologue from *Corynebacterium bovis* was found to expose only a single surface lysine. However, this aldolase did not show increased stability towards acetaldehyde, compared to DERA_EC_ (see ESI, Fig. S1†). Thus, a reaction of acetaldehyde with surface lysines could be demonstrated, but it does not trigger deactivation of the enzyme.

### Investigation of acetaldehyde effects by NMR spectroscopy

Until now, efforts to understand the mechanism behind acetaldehyde-related DERA deactivation have suffered from missing structural information on the enzyme after incubation with the substrate. In order to fill this gap, DERA was first analyzed *via* NMR spectroscopy, which is highly suitable and sensitive to detect any kind of alteration related to, *e.g.*, unfolding, conformational changes, or substrate binding, *via* changes in chemical shifts.^19^

**Fig. 2:**
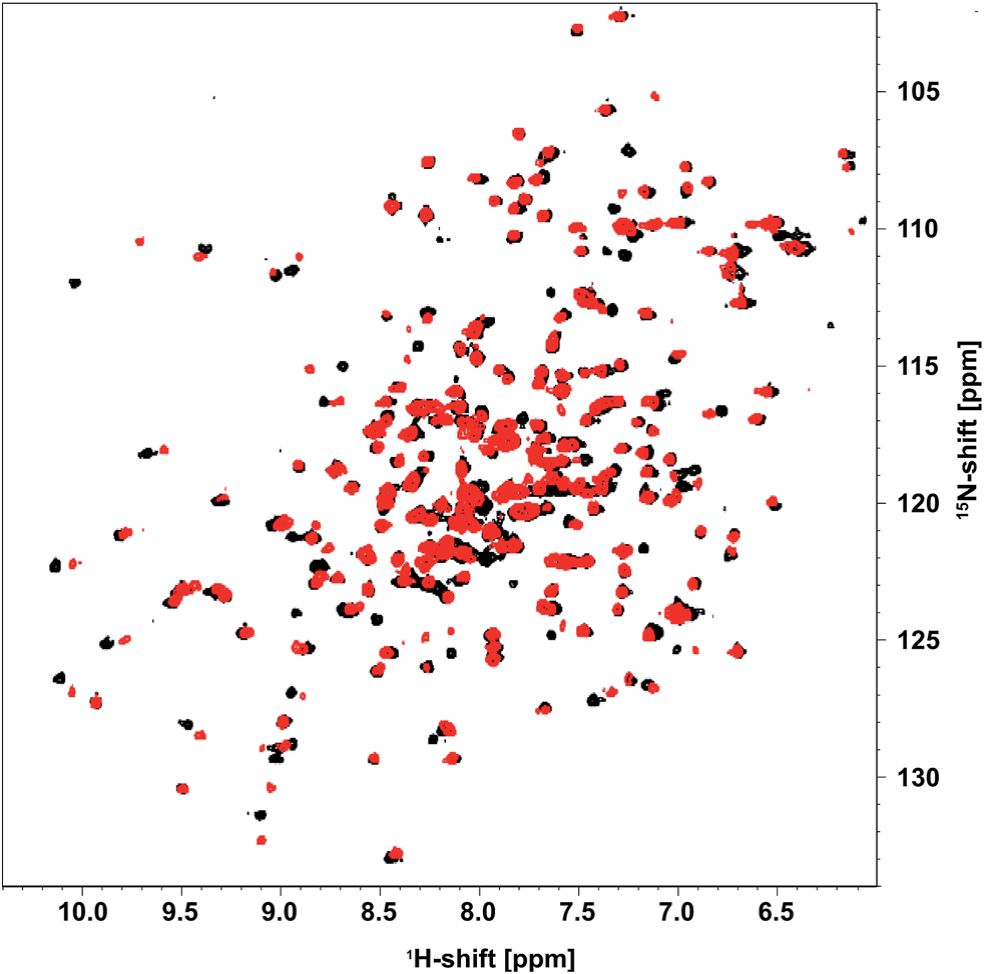
[^1^H,^15^N]-TROSY spectrum of [*U*-^15^N] monomeric DERA recorded before (black) and after (red) treatment with acetaldehyde.

As DERA is a dimeric protein, it yields broader resonance peaks than the respective monomer, with reduced spectral resolution due to signal overlap. Hence, a monomeric variant of the enzyme (K58E-Y96W) was used that has virtually identical biochemical properties.^20^ Apart from the two exchanges, the X-ray structure of this monomerized DERA (see below) is practically identical to individual chains of the wildtype protein (PDB-ID 1JCL), with an overall root-mean-square distance of 0.38 Å for all resolved main-chain atoms. This observation supports the utility of the double mutant for structural investigations. A [^1^H,^15^N]-TROSY spectrum of the monomeric DERA was recorded before and after acetaldehyde incubation (Fig. 2). Most signals remained unchanged, which is an indication that the overall structure of the protein is unaltered after acetaldehyde treatment. Nonetheless, direct comparison reveals differences in chemical shifts for a distinct set of resonances.

In order to identify the affected residues, we have assigned the backbone resonances of the untreated, [*U*-^13^C,^15^N]-DERA monomer using HNCA, HN(CO)CA, HNCO and HN(CA)CO experiments. Information about C_β_ atoms of the sidechains could not be obtained *via* HNCACB and HN(CO)CACB triple resonance experiments due to a low signal-to-noise ratio (probably related to the size of the protein).^21^ Instead a 3D [^1^H,^1^H,^15^N]-TOCSY-HSQC experiment was performed, where magnetization is transferred between the ^1^H spins of the sidechains and then *via* the ^15^N nuclei to the H_N_-protons for detection.^22^ This approach enabled us to detect two signals at around 4 and 1.5 ppm for the H_β_ and H_α_ nuclei of the alanine residues. With this information at hand it was possible to assign all amino acids except the terminal segments and S239, which is located in a loop region (in total 95% of the protein sequence was assigned). The assigned TROSY spectrum is shown as ESI (Fig. S2†), and the chemical shifts have been deposited in the *Biological Magnetic Resonance Data Bank* (accession number 25904). For quality control the secondary structure of the protein was determined with CSI 2.0 (ref. 23) using the chemicals shifts and then compared with the X-ray structure. Furthermore, our experimental chemical shifts were plotted against the theoretical values that were calculated by SPARTA,^24^ based on the 3D-structure (PDB-ID 1JCL). The results revealed a high correlation between experimental and calculated values and are available as ESI (Fig. S3 and S4†).

**Fig. 3:**
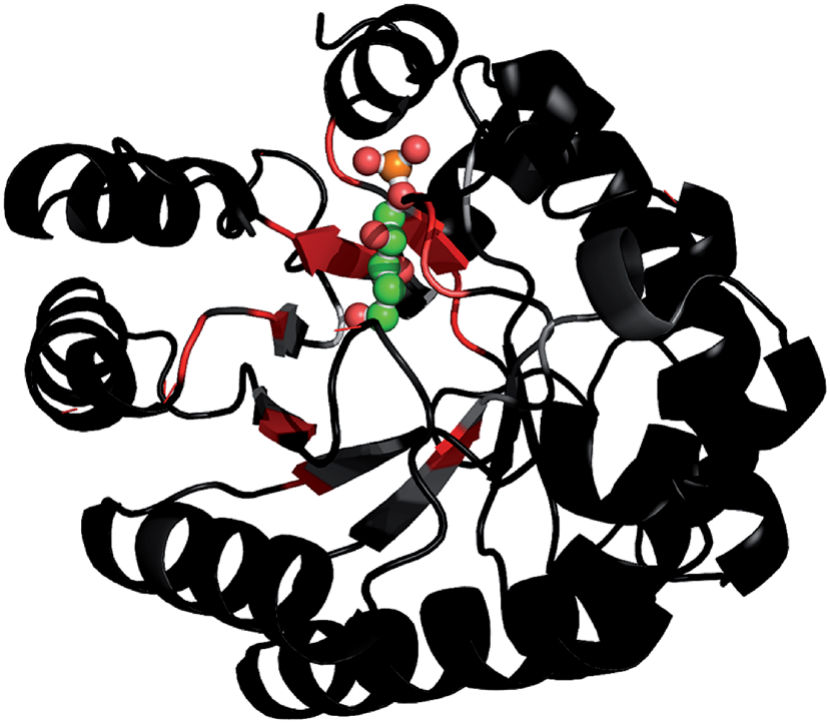
After the backbone assignment of DERA monomer, the chemical shift perturbation 
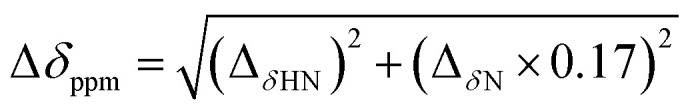
 between both states was calculated and mapped to the X-ray structure. The differences vary from black (0 ppm) to red (>5 ppm). The natural substrate (2-deoxy-d-ribose-5-phosphate, adopted from PDB-ID 1JCL) is represented in CPK mode (phosphorus in orange, oxygen in red and carbon in green).

Based on the backbone assignment, the chemical shift perturbation, quantifying the combined chemical shift differences in 2-dimensional NMR spectra was calculated according to Tochio *et al.*^25^ for all resonances before and after acetaldehyde incubation (Fig. 3). Alterations in chemical shifts were found exclusively in the inner part of the protein, mainly in the area where the natural substrate would bind. This finding lead to the conclusion that after the washing step acetaldehyde and/or reaction products thereof remain in the substrate pocket and somehow block the active site of the protein.

### Identification of reaction products

**Fig. 4:**
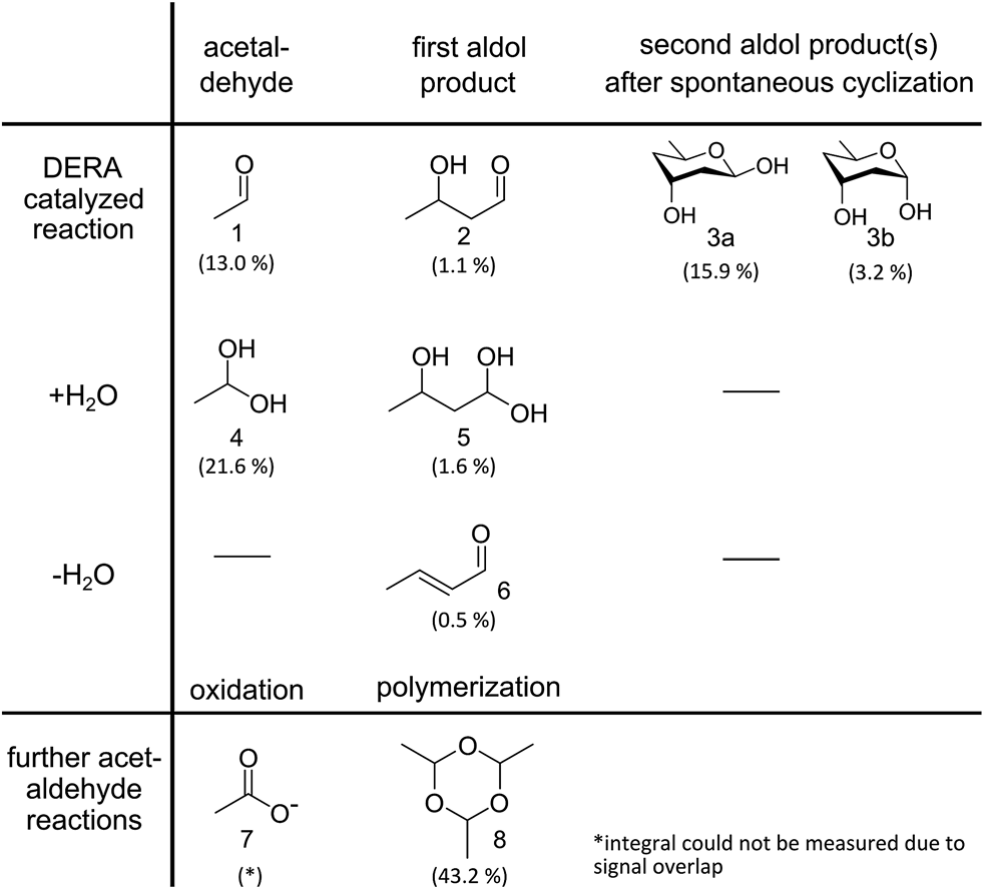
Reaction products identified *via* NMR analysis after overnight incubation of DERA with ^13^C-labeled acetaldehyde. A detailed analysis of the spectra is provided as ESI† Methods. Percentages indicate the molar fraction of each molecule, derived from the integrals of the proton spectrum, in relation to all species where such integrals could be determined. A negative control (acetaldehyde in water without enzyme) leads only to compounds 4, 7 and 8, the formation of which does not involve an aldol reaction.

DERA catalyzes a sequential aldol reaction, resulting in addition products formed out of two or three acetaldehyde molecules. The second product is converted into a lactol by spontaneous cyclization.^26^ Furthermore, the addition or elimination of water molecules increases the diversity of possible reaction products. Investigation of soluble compounds formed after reaction of DERA with acetaldehyde could help identify candidates that might block the binding pocket of the enzyme and would also provide information on side products that appear during the reaction. As separation of the reaction products is hardly possible due to their instability, a DERA sample was incubated over night with ^13^C-labeled acetaldehyde, followed by removal of the protein and NMR analysis of the aqueous product mixture using [^1^H,^13^C]-ctHSQC, INADEQUATE (CC–COSY), [^1^H–^1^H]-TOCSY and [^1^H–^1^H]-COSY experiments. The assignment was supported by a recently published NMR study on acetaldehyde in aqueous solution.^27^ An overview of all detected compounds and their possible reaction pathways is given in Fig. 4.

**Fig. 5:**
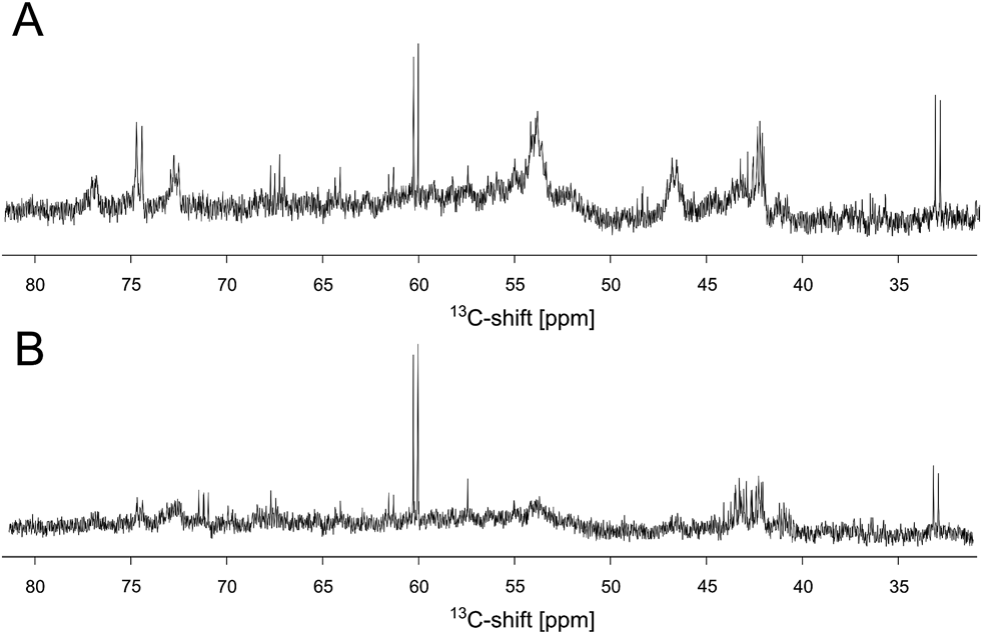
Extract of the ^13^C spectra of unlabeled DERA after incubation with ^13^C-labeled acetaldehyde. (A) 1D spectrum recorded after removal of free reaction products. (B) The same sample after a heating step of 4 h at 50 °C. A negative control of DERA without substrate incubation (not shown) was used to exclude misinterpretation of signals due to the natural abundance of ^13^C within the protein.

In order to investigate whether these reaction products bind to the protein, an unlabeled DERA was incubated over night with 300 mM [*U-*^13^C]-acetaldehyde, and after several extensive washing steps a ^13^C-NMR spectrum of the enzyme was recorded (Fig. 5A). Various peaks clearly signify the presence of ^13^C-labeled compounds. While sharp signals (*e.g.* at 60 and 33 ppm) indicate that there are still free aldol products in solution (probably caused by an equilibrium between a protein-bound and an unbound state), some broad signals appear at 55 and 42 ppm, suggesting association with the protein. The whole spectrum is given as ESI (Fig. S5†), together with an assignment which was assisted by later findings on possible products and their binding mode within the protein (see below). To further validate this conclusion, iDOSY-ctHSQC measurements were performed, which can be used to determine the diffusion coefficients (related to the molecular mass) of the ^13^C-labeled compounds (ESI, Fig. S6†). Thereby it was possible to show that there are indeed ^13^C atoms diffusing with the enzyme, as signals appear in the region of macromolecules.

### Reactivation of DERA *via* heating

**Fig. 6:**
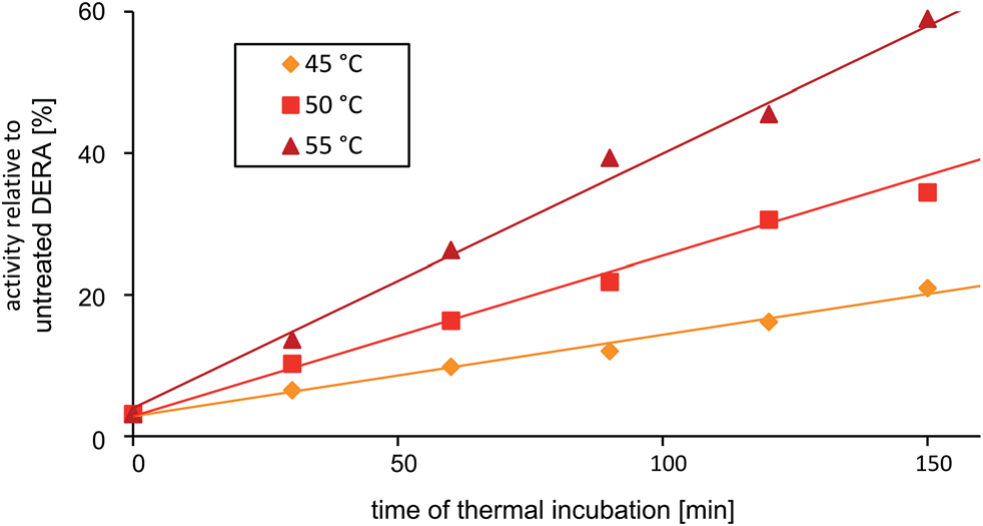
Partial reactivation of DERA (dimeric wildtype) after acetaldehyde incubation *via* heating at various temperatures.

The discovery that product(s) of the DERA catalyzed reaction appear to interact with the protein and may thus block the binding pocket prompted us to search for strategies to overcome this deactivation. Specifically, we tested if enzyme activity could be recovered by means of heating, as the dimeric DERA is rather thermostable and remains fully active after several hours of incubation at 55 °C (50 °C in case of the monomeric variant).^20^ The results are shown in Fig. 6 and demonstrate that heating is indeed a way to regain enzyme activity after acetaldehyde incubation. Overall, up to 70% of wildtype activity could be recovered for the dimeric enzyme after an incubation time of >6 h at 55 °C. Similarly, about 50% activity is detected for the monomeric mutant after >4 h at 50 °C (data not shown). This partial reactivation was further investigated by NMR. On the protein level, heating of [*U*-^15^N]-DERA after acetaldehyde treatment caused distinct changes in the chemical shifts (see ESI, Fig. S7†): while some resonance frequencies remained the same as after acetaldehyde incubation, others reverted to their original values (*i.e.*, those prior to incubation). In addition, new signals appeared. In some cases two states reflecting the situation before and after acetaldehyde treatment, respectively, appeared to coexist. The spectrum can be interpreted as an equilibrium or mixture between the active and inactive DERA variants, which is in agreement with the kinetic measurements of the monomeric aldolase showing 50% of the initial activity after heating. Subsequently, the ^13^C spectrum of DERA incubated with ^13^C-labeled substrate was recorded again after the heating step. In Fig. 5, corresponding regions of both 1D spectra are compared, showing a reduced area of the broad peaks after heating, which is probably caused by the removal of ^13^C-labeled molecules from the protein. As expected, the DOSY spectrum (given as ESI, Fig. S6†) also indicates a partly removal of ^13^C atoms from the protein after heating.

### Covalent modification of DERA after acetaldehyde incubation

**Fig. 7:**
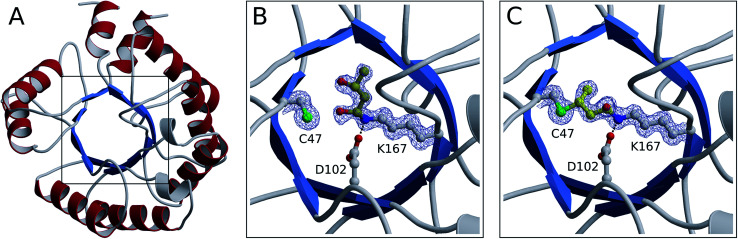
Crystal structures of monomerized *E. coli* DERA. An overview of the fold is shown in ribbon representation (A). The boxed area containing the catalytic center is depicted in more detail before (B) and after (C) acetaldehyde incubation. Amino acid side chains as well as covalently bound substrates are displayed in ball-and-stick mode (nitrogen in blue, oxygen in red, sulfur in green, carbon in light gray and yellow for amino acids and substrates, respectively). In both structures, electron density is contoured at 1 σ for compounds involved in covalent linkage.

From our NMR studies we conclude that product(s) derived from the DERA-catalyzed reaction bind to the inner part of the protein, resulting in an inactive enzyme. To determine the structure of these reaction product(s) and to identify the exact target site(s) within the protein, X-ray structures of DERA from *E. coli* before and after acetaldehyde incubation as well as after thermal treatment have been determined (Fig. 7). Crystals were obtained for all three states under the same conditions, yielding datasets with resolutions ranging from 1.1 Å (native enzyme) to 1.5 Å (after heating). X-ray data collection and refinement statistics are provided as ESI, Table S1.†

All three structures turned out to be very similar, with obvious alterations limited to the catalytic center. Interestingly, the native enzyme features electron density for a six-atom molecule with about 70% occupancy, which is attached to the active lysine residue (Fig. 7B). The covalent structure of this ligand is consistent with an aldol product formed by two acetaldehyde molecules (compound 2 in Fig. 4), which forms a hemiaminal (a hydrated Schiff base) with the lysine amino group. Given that this type of linkage is expected from the well-established catalytic mechanism of DERA, our findings may be rationalized by the presence of trace amounts of aldehydes in bacterial lysates or crystallization solutions. Importantly, this appears to be a reaction intermediate reflecting the intrinsic aldolase activity of the protein. However, after treatment with an excess of acetaldehyde (300 mM), a very different situation is observed (Fig. 7C): the ligand has changed its orientation and established a covalent link with the sulfur atom of a nearby cysteine residue (C47). Note the absence of the distal branch in the ligand structure, which probably reflects loss of a hydroxyl group; a likely mechanism leading to the formation of this product is discussed below. The relative stability of the thioether adduct provides a plausible explanation for the irreversibility of DERA inhibition by acetaldehyde at customary temperatures.

Subsequent heating at 50 °C results in further (albeit more subtle) changes in the crystal structure. Specifically, we observe a reduction in occupancy of the bridging ligand (70%, compared to 87% prior to heating), which is accompanied by the appearance of a weak difference density possibly indicating partial displacement of the inhibitor (not shown). Together, these observations strongly suggest that acetaldehyde at high concentrations or, more specifically, the formed homoaldol adduct acts as a mechanism-based inhibitor of DERA. At increased temperatures, the peculiar thioether link undergoes time-dependent elimination, accounting for gradual recovery of enzyme activity.

### Deactivation mechanism

**Fig. 8:**
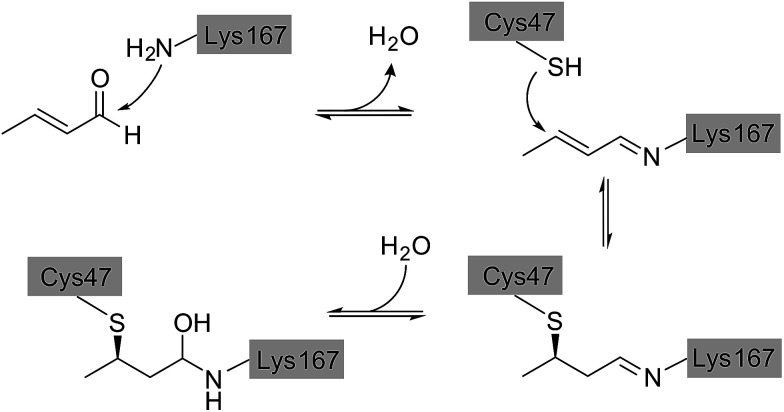
Proposed reaction mechanism leading to DERA inactivation. Crotonaldehyde forms an imine with the amino group of K167, followed by Michael addition of C47. Hydration of the double bond leads to the final compound that was found in the crystal structure of the enzyme after acetaldehyde incubation.

Direct comparison of the DERA crystal structures described above reveals two major changes occurring in the K167-bound aldol product upon acetaldehyde incubation: first, the hydroxyl group at the C_β_ atom is missing, and second, the substrate has formed a covalent bond to C47. Our NMR studies have demonstrated that 3-hydroxybutanal is prone to water elimination, resulting in loss of the hydroxyl group and formation of the respective α,β-unsaturated aldehyde (see Fig. 4, compounds 2 and 6). As shown in Fig. 8, the reaction product, crotonaldehyde, can now react as a substrate to form a Schiff base with K167. In a second step, the thiol group of C47 acts as nucleophile and attacks the sp^2^ hybridized C_β_ atom of crotonaldehyde. Indeed, incubation of DERA with this particular compound yielded more than 100-fold stronger inhibition, compared to acetaldehyde; specifically, a concentration as low as 1 mM leads to virtually full inactivation within less than one hour (see ESI, Fig. S8†). This finding supports the proposed mechanism of DERA deactivation by this mechanism-based inhibitor.

It should be noted that, based on electron density alone, we cannot strictly exclude a reverse orientation of the covalently bound ligand. In an alternative reaction mechanism, the nitrogen atom of K167 might act as a nucleophile for Michael addition to crotonaldehyde, while the cysteine residue could form a hemithioacetal with the aldehyde group (see ESI, Fig. S9†). Similarly, the ^13^C chemical shifts recorded after DERA incubation with ^13^C-labeled acetaldehyde (Fig. 5) are compatible with either mechanism (ESI, Fig. S5†). Nevertheless, in view of the natural catalytic pathway of DERA the second pathway seems less likely as the enzyme is optimized to form a Schiff base of the aldehyde moiety with the amino group of the catalytic lysine. Further indications against this alternative reaction mechanism were found when solving the X-ray structure of DERA after incubation with 5 mM of (*E*)-2-pentenal. The initial idea was to determine the orientation of the molecule linking C47 and K167 based on the additional carbon atom, compared to crotonaldehyde [(*E*)-2-butenal]. However, the larger compound turned out not to form a covalent bond with C47. Our crystal structures indeed suggest that the bulky ethyl substituent cannot be easily accommodated by the pocket lined by D16, T18, C47, and K201, which may prevent Michael addition of the thiol to the C=C bond. Notably, such steric hindrance would not appear relevant for the alternative orientation, where the ethyl moiety would be facing the substrate channel.

### Acetaldehyde resistant enzyme variant

Since the catalytic K167 cannot be exchanged without losing the enzyme function, a viable strategy to prevent this irreversible deactivation might be mutation of the C47 residue. A sequence alignment of *E. coli* DERA with 3700 orthologues (≥20% sequence identity) showed a frequency of 91% for cysteine at position 47. Thus, although being highly conserved, C47 is not essential for catalytic activity.^5^

The critical cysteine has been replaced by methionine, a rather similar but non-nucleophilic amino acid, by means of site-directed mutagenesis. Biochemical characterization of the new variant showed that it retains 60 ± 5% of the wildtype activity towards its natural substrate, and the melting temperature of 65.1 °C suggest a similar thermostability as found for wildtype DERA (66.6 °C). Both proteins were incubated with different concentrations of acetaldehyde and the activity was recorded as a function of time.

**Fig. 9:**
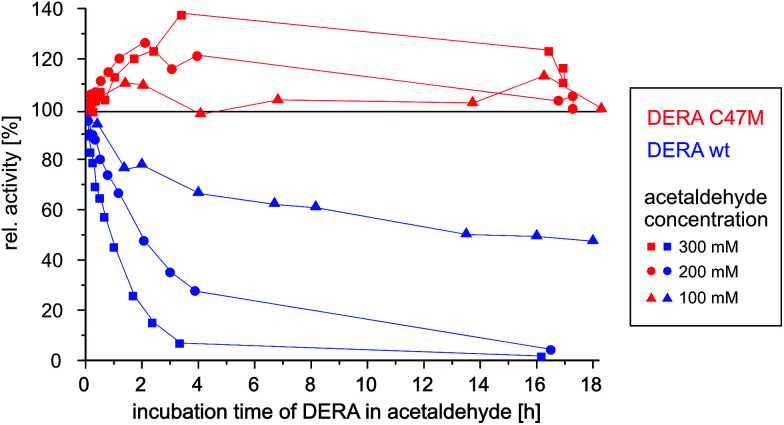
Time-dependent activity of wildtype *vs.* C47M DERA during incubation at different acetaldehyde concentrations.

As can be seen in Fig. 9, wildtype DERA is completely inactive after 3 h of incubation with 300 mM acetaldehyde, while at 100 mM an activity of around 50% remains after 16 h. In contrast, the C47M mutant did not lose any activity even after 16 h in the presence of 300 mM acetaldehyde. Further tests with 1 M of the substrate yielded the same result (see ESI, Fig. S10†). Surprisingly, apparent activity even tends to increase, which may be due to altering reaction mixture compositions and their influence on the enzyme (see Experimental section). Irrespective of this aspect, however, the data clearly demonstrate the resistance of the C47M mutant towards inactivation by acetaldehyde.

These results provide further support for the inactivation mechanism depicted in Fig. 8 and also suggest an easy solution to the problem: by replacing a reactive residue, *i.e.*, a single point mutation, we were able to create a fully acetaldehyde resistant *E. coli* DERA.

It is interesting to note that already in 1976, a long time before any structural information on this enzyme was available, D. C. Wilton identified acrolein (propenal) as an irreversible inhibitor of DERA and proposed an inactivation mechanism where first a Schiff base is formed between the aldehyde and the catalytic lysine and subsequently an unknown nucleophile forms a covalent bond with the Michael system.^28^ Our studies indicate that this nucleophile is C47 and acts in the same way for crotonaldehyde [(*E*)-2-butenal], which is formed upon water elimination from the aldol product between two acetaldehyde molecules.

### Catalytic properties of the C47M variant

**Fig. 10:**
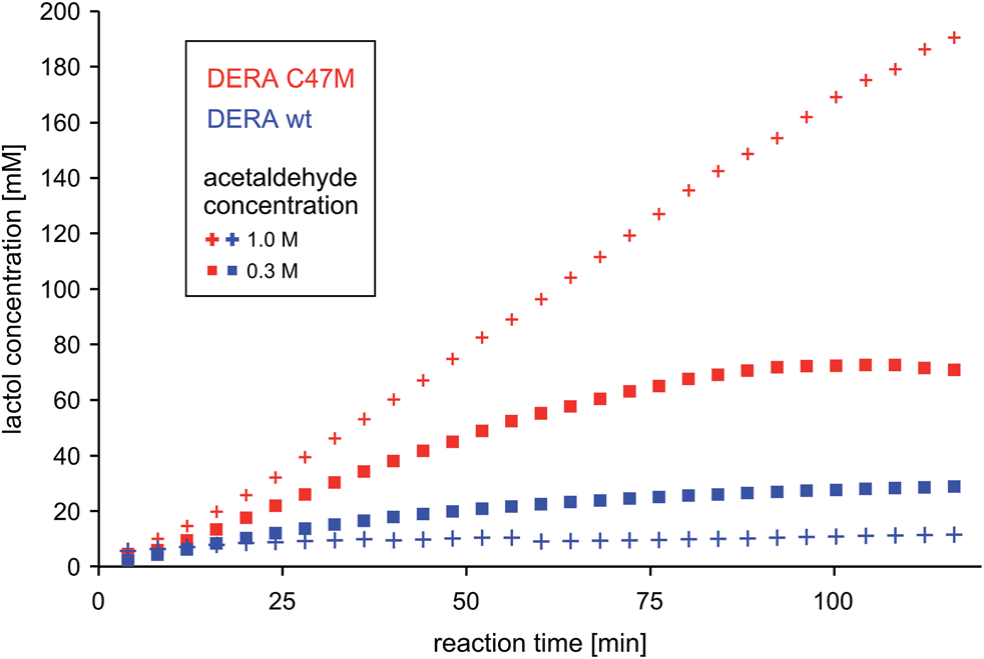
Time-dependent lactol (compound 3 in Fig. 4) formation by wildtype DERA *vs.* its C47M mutant. Product concentrations were determined *via* integration of ^1^H-signals in the NMR spectra. More detailed analyses regarding substrate conversion and formation of the single aldol product are given as ESI, Fig. S11.†

To test the potential of the new mutant in organic synthesis, reaction kinetics of both wt and C47M DERA were assessed by NMR spectroscopy in 300 mM and 1 M acetaldehyde. As can be seen in Fig. 10, there is a clear difference in product formation between both enzyme variants. In the presence of wt DERA only small amounts of lactol are synthesized in 300 mM of acetaldehyde, while at a higher substrate concentration almost no product can be detected (likely caused by very fast deactivation of the enzyme). On the contrary, the C47M mutant leads to faster and sustained product formation, until the reaction equilibrium between acetaldehyde and its single and double aldol products is reached.

To validate if this new mutant is still as versatile a catalyst as the wildtype DERA, we performed a (qualitative) HPLC-MS analysis of DERA-catalyzed reactions using acetaldehyde as donor and various acceptor aldehydes, which revealed the same pattern of the respective single and double aldol products as found with wildtype DERA (Table 1). Indeed the mutant performed even better than the wildtype with respect to the reaction of crotonaldehyde.

**Table 1:** HPLC-MS analysis of DERA-catalyzed aldol reactions using various acceptor aldehydes. Products were detected after derivatization with 2,4-dinitrophenylhydrazine

Acceptor	Single aldol		Double aldol	
	WT	C47M	WT	C47M
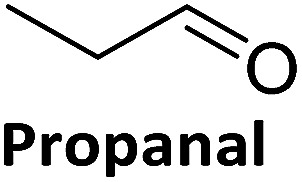	+	+	+	+
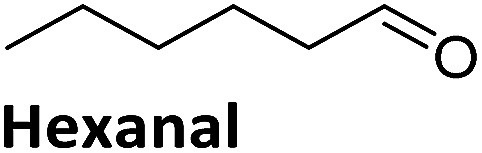	+	+	+	+
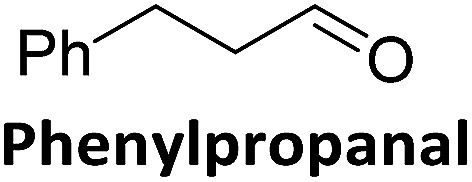	+	+	+	+
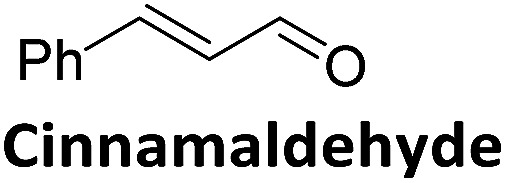	−	−	−	−
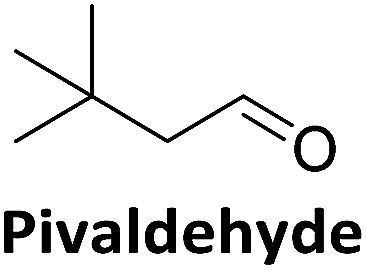	+	+	+	+
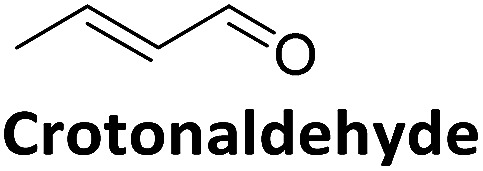	−	Traces	Traces	+

### Acetaldehyde tolerance of DERAs from different organisms

Many different DERAs from various organisms have been described in the literature showing a higher acetaldehyde tolerance compared to wildtype DERA from *E. coli* (see Introduction). However, a sequence alignment revealed the same critical cysteine at position 47 – except for one example of *Hyperthermus butylicus* (unknown structure)^12^ – that may cause the nucleophilic attack. Hence, the higher acetaldehyde tolerance of other DERAs cannot be explained by a different amino acid at position 47. As mentioned above, there might be a correlation between thermostability and acetaldehyde tolerance because most of the new enzymes came from thermophilic organisms.^11,17^ Due to their adaptation to high temperature, they show a lower rate of substrate conversion and thus produce a lower amount of crotonaldehyde. Further experiments with *E. coli* DERA suggest a relationship between the half-life of that enzyme in acetaldehyde and the amount of protein used in the assay (the higher the protein amount the higher the substrate tolerance, see ESI, Fig. S12†). This effect impedes direct comparison of published results regarding acetaldehyde stability of different DERAs, as enzyme concentrations are often not reported. In addition, the environment and the distance between C47 and the C_β_-atom of crotonaldehyde can influence the deactivation process. All three points might contribute to the higher acetaldehyde tolerance that was detected in other DERAs.

The relative conservation of C47 raises the question of its specific function. Mechanistic studies from Heine *et al.* have shown that it is not essential for the catalytic mechanism.^5^ However, mutations lead to a less active DERA, in accordance with our studies. It is important to recall that in its physiological setting, DERA chiefly catalyzes a retro-aldol reaction, in which acetaldehyde occurs as a product. Since acetaldehyde is harmful to the cell, a suicide (mechanism-based) inhibition at high concentrations of this compound might thus act as a protective mechanism by terminating the conversion of deoxyribose-5-phosphate to glyceraldehyde-3-phosphate and acetaldehyde.

### Mechanism-based inhibition in nature and biocatalysis

Covalently binding inhibitors, often effecting mechanism-based (or suicide) inhibition, have evolved as an important class of compounds regulating enzyme activity; a particularly well-understood case is the superfamily of serpins, which are involved in downregulation of proteases.^29^ For clinical and pharmaceutical applications, numerous drugs have been developed that function as suicide inhibitors. Prominent examples are aspirin (acetylation of serine in prostaglandin-endoperoxide synthase),^30^ penicillin (acylation of serine in dd-transpeptidase)^31^ and α-difluoromethylornithine (DFMO), a compound targeting African trypanosomiasis (sleeping sickness), which covalently binds to pyridoxal phosphate in ornithine decarboxylase and is subsequently transferred to a cysteine residue, thus deactivating the enzyme.^32^

On the other hand, mechanism-based inhibition as an unwanted side reaction in biocatalysis is rarely described in literature. While studies on optimizing biocatalysts towards high operating temperature or stability in organic solvents are published regularly^33–36^ the role of substrates and reaction intermediates/products has been investigated in few cases only. Franken *et al.* analyzed the role of acetaldehyde as the product of the lipase-catalyzed reaction between vinyl esters and alcohols. Using mass spectrometry they were able to identify aldol condensation products such as crotonaldehyde, which form Michael adducts with surface-exposed amino acids.^37^ A role of cysteine residues in irreversible substrate inhibition has been described for the reduction of *S*-nitroglutathione by the human carbonyl reductases 1 and 3.^38^ When using a high substrate concentration, C227 forms a covalent bond with the sulfur atom of glutathione, which can be removed only under reducing conditions. In line with our results, these examples reveal a fundamental issue in biotechnology: when used in organic synthesis, enzymes necessarily operate in non-native environments, increasing susceptibility to unwanted side reactions. Hence, there is a need for individual adaptation of biocatalysts to overcome such problems. Mass spectrometry, NMR and X-ray crystallography, particularly when used in combination, are able to provide a detailed account of toxic compounds and their reaction products with the protein of interest. Rational protein design based on this knowledge is a viable strategy to improve the biocatalyst for use in organic reactions.

## Conclusions

In this study, we have identified crotonaldehyde, the product of aldol condensation between two acetaldehyde molecules, as a mechanism-based inhibitor of DERA. This substrate acts by covalently linking the catalytic lysine residue to a nearby cysteine. To our knowledge, this is the first detailed account of mechanism-based inhibition by a reaction product derived from a natural substrate in biocatalysis. Ultimately, these findings are related to the more general challenge of using this reactive substrate (and probably other aldehydes) in synthesis, involving many side reactions and possible binding of the products to the enzyme. In the case of DERA, an elegant solution is achieved by replacing a single reactive residue, yielding an acetaldehyde-resistant enzyme. We are confident that our results will inspire similar efforts to improve the performance of enzymes in biotechnology applications.

## Experimental section

### Chemicals

All chemicals and enzymes, if not mentioned separately, were purchased at Carl Roth (Karlsruhe), Sigma Aldrich (Steinheim) or (for labeled compounds) at the Cambridge Isotope Laboratories (Saarbrücken) in analytical grade.

### Cloning and protein expression

DERA-coding *deoC*-gene from *C. bovis* (codon harmonized) was synthesized by GenScript and cloned into the *pET21a*-vector *via Nde*I and *EcoR*I restriction sites. The *deoC*-gene from *E. coli* had been isolated previously.^10^ Both genes contained a coding region for a C-terminal 6 × His-Tag. The C47M mutation was inserted *via* round-the-horn^39^ mutagenesis with 5′-phosporylated ATGATCTATCCTCGCTTTATCCCGATTGC as forward and GATAGCGGCGGTATTGCCGACC as reverse primer using Phusion DNA-polymerase (Stratagene) and verified by sequencing (GATC Biotech). Enzymes were expressed in *E. coli* BL21(DE3) strain in TB-medium. For ^15^N- and/or ^15^N–^13^C-labeled proteins M9 minimal media according to Sivashanmugam *et al.*^40^ were used. Expression was initiated by addition of 0.1 mM IPTG, and after 16 h incubation at 25 °C cells were harvested by centrifugation (15 min at 7000 × *g*).

### Sample preparation for kinetic and structural studies

For enzyme purification 1–4 g cells were resuspended in 5 volumes of 20 mM potassium phosphate (KP_i_)-buffer at pH 7.0, disrupted *via* sonification, and after centrifugation (15 min at 12 000 × *g*) the supernatant was loaded onto a NiNTA-column in a cyclic process (15 min). After washing with KP_i_-buffer containing 30 mM imidazole the enzyme was eluted with 250 mM imidazole. PD-10 desalting columns were used to rebuffer the solution to 20 mM KP_i_, and purity (≥95%) was verified with SDS-PAGE. The protein concentration was determined by the Bradford assay.^41^ Assessment of protein stability by means of CD spectroscopy has been described previously.^20^

For NMR studies the pH of the solution was adjusted to 6.8, the protein was concentrated to 0.2–0.4 mM and 5% D_2_O was added. The protein concentration was further increased to 1 mM for 3D NMR measurements and 0.05% NaN_3_ was added. Prior to NMR and crystallization screening experiments with acetaldehyde-treated DERA, the substrate and soluble products were removed *via* PD-10 desalting columns (2×). ^13^C-labeled reaction products were separated from the protein using a centrifugal filter device (VivaSpin). For NMR kinetics, a solution of 2 mg mL^−1^ protein in 120 mM KP_i_ pH 7 including 5% D_2_O was prepared. Directly before measurement a solution of acetaldehyde in 100 mM KP_i_ pH 7 was added, resulting in a final concentration of 300 mM and 1 M, respectively. The measurements were performed over 2 h.

Prior to crystallization screening a second purification step *via* size exclusion chromatography was performed. A SuperdexTM200 10/300 GL (GE Healthcare) column was equilibrated in 100 mM KPi, 150 mM NaCl (pH 7); 5 mg protein was loaded on the column, and the elution profile was recorded by means of absorbance at 280 nm.

### Kinetic measurements

The activity for cleavage of the natural substrate 2-deoxy-d-ribose-5-phosphate (DRP) was measured for 1 min at 25 °C. In a retro-aldol reaction, glyceraldeyhde-3-phosphate is formed. The latter is reduced to glycerol-3-phosphate by the auxiliary enzymes glycerol-3-phosphate dehydrogenase (GDH) and triose phosphate isomerase (TPI) under NADH consumption, which was detected with a photometer at 340 nm.^42^ The standard reaction mix (400 μL) contained 0.4 mM DRP, 0.15 mM NADH, 4 U GDH, 11 U TPI and 10 μL DERA solution. To determine the stability towards acetaldehyde, 0.1–1 M of freshly distilled substrate (aged acetaldehyde solutions led to inconsistent results) was added to the enzyme solution (2 mg mL^−1^); the first kinetic measurement was performed immediately after mixing. Additional samples were taken at regular intervals and their activities determined. Note that the samples contained both enzyme and acetaldehyde used for pre-incubation. This aldehyde may function as product inhibitor for the retro-aldol reaction which is exploited for activity measurement. As a consequence, product inhibition will become the weaker the more acetaldehyde is converted to an aldol product by an active DERA, possibly explaining the time-dependent increase in activity of the C47M mutant.

### HPLC-MS analysis

The aldol reactions were performed on an analytical scale according to a protocol established in our laboratory.^20^ The catalysts were used as lyophilized protein (wildtype DERA_EC_: 2.2 mg with 8.3 U mg^−1^, C47M mutant: 4.4 mg with 5 U mg^−1^), dissolved in 0.5 mL of triethanolamine buffer (0.1 M, pH 7). Each acceptor aldehyde was used with equal substance amount (0.10 mmol). The extracted and dried samples were dissolved in acetonitrile. HPLC-MS measurements were performed on an Agilent1100 HPLC system equipped with a C18 Eclipse Plus column (100 × 4.6 mm, 2.6 μm particle size [Agilent Technologies], operated at 40 °C) interfaced with a Q-TRAP 4000 (ABSciex). As solvent a mixture of (A) H_2_O with 0.1% formic acid and (B) acetonitrile with 0.1% formic acid was used, applying a flow rate of 0.8 mL min^−1^ and the following gradient: 60% A – 2 min, then 10% A – 25 min. The particular product mass traces were extracted.

### Mass spectrometry of DERA

DERA (6 mg mL^−1^) was incubated for 3 h with 300 mM acetaldehyde in 200 mM sodium borate buffer, pH 8.5, followed by reduction in sodium borohydride (400 mM) to stabilize the Schiff bases.^43^ The protein was precipitated with 10% trichloroacetic acid, washed with acetone, dissolved in 100 μL Tris–HCl buffer (100 mM, pH 8.5) containing 8 M urea and diluted to 400 μL and 2 M urea in Tris–HCl. After a tryptic digest (1 h with 0.125 mg mL^−1^ trypsin) the protein sample was desalted by RP C4 ZipTips (Millipore) and analyzed by LC-MALDI-TOF/TOF mass spectrometry (Ultraflextreme; Bruker Daltonics).

### NMR studies

NMR experiments were conducted at 25 °C on Bruker Avance III HD (600 and 700 MHz), Bruker Avance/DRX 600 (600 MHz) and Varian VNMRS (900 MHz) instruments, equipped with cryogenic *Z*-axis pulse-field-gradient (PFG) triple resonance probes. For ^1^H and ^13^C chemical shifts 4,4-dimethyl-4-silapentane-1-sulfonic acid (DSS) was used as internal standard at 0 ppm. ^15^N chemical shifts were referenced indirectly using frequency ratios.^44^ For NMR kinetics at Bruker Avance/DRX 600, ^1^H measurements were recorded relative to the resonance of the solvent. Assignment of protein backbone resonances was performed using a combination of multidimensional NMR experiments: 2D [^1^H–^15^N]-TROSY,^45^ 3D HNCA,^46^ 3D HN(CO)CA,^47^ 3D HNCO,^48^ 3D HN(CA)CO^49^ and 3D [^1^H–^1^H–^15^N]-TOCSY-HSQC^22^ experiments at 900 MHz. Raw data for backbone assignment were processed with NMRPipe v.8.1 (ref. 50) and analyzed with CppNmr v.2.4.^51^

### Protein crystallization and diffraction data collection

Samples of monomeric *E. coli* DERA (K58E-Y96W mutant) were subjected to crystallization screening at 20 °C in a sitting-drop setup, using a robotic system (Freedom EVO, Tecan). Crystals were observed for a number of conditions, including reservoir solutions containing 10% (w/v) PEG4000, 20% (v/v) 2-propanol (native enzyme), or 20% (w/v) PEG4000, 10% (v/v) 2-propanol, 0.1 M HEPES pH 7.5 (all samples), and protein concentrations of 10–20 mg mL^−1^. Prior to cryocooling, crystals were soaked in reservoir buffer containing 7–10% (v/v) glycerol. Single-wavelength diffraction datasets were recorded at 100 K on beamlines ID23-1 and ID29 of the European Synchrotron Radiation Facility (ESRF; Grenoble, France) equipped with PILATUS 6M and PILATUS3 6M detectors (Dectris), respectively. Data processing was carried out using XDS and XSCALE.^52^

### Structure determination

Crystals of monomeric *E. coli* DERA belonged to space group *C*2. Initial phases for the native enzyme were obtained by molecular replacement with MOLREP,^53^ using the wildtype structure (PDB-ID 1JCL) as template. The crystals contain a single chain per asymmetric unit, corresponding to a Matthews coefficient of 1.9 Å^3^ Da^−1^ and a solvent content of 37%. The initial model was improved iteratively by alternating reciprocal space refinement in phenix.refine^54^ with manual rebuilding in Coot.^55^ Structures of acetaldehyde-treated DERA before and after heating were refined starting from the structure of the native protein. Validation with MolProbity^56^ revealed good geometry in every case, with all of the residues in the allowed regions of the Ramachandran plot. Statistics on data collection and refinement are provided as ESI, Table S1.† Atomic coordinates and structure factor amplitudes have been deposited in the Protein Data Bank with accession codes 5EKY (native monomerized DERA), 5EL1 (after acetaldehyde treatment) and 5EMU (after additional heating).

### Molecular graphics

Ribbon representations in Fig. 7 were generated with POVScript+^57^ and Raster3D,^58^ applying secondary structure assignments provided by DSSP.^59^

## Author contributions

M. D., T. C., P. N. and J. P. conceived the project; M. D. cloned, purified and characterized the enzymes; R. H. and M. D. performed the NMR studies with assistance from M. S. for the backbone-assignment. C. B. with support from R. H. and M. D. measured NMR kinetics; O. H. W. with assistance from M. D. and T. C. crystallized the enzymes and determined the structures; C. B. performed the biotransformations; the manuscript was drafted by M. D. and finalized with contributions from all authors; D. W. and J. P. supervised research.

## Supplementary Material

SC-007-C5SC04574F-s001
