# Supplementary material for: Mechanism-based inhibition of an aldolase at high concentrations of its natural substrate acetaldehyde: structural insights and protective strategies
Source: Chem Sci. 2016 Mar 30;7(7):4492–502. doi: 10.1039/c5sc04574f (PMC6016325; doi:10.1039/c5sc04574f)
Supplement: SC-007-C5SC04574F-s001 [file SC-007-C5SC04574F-s001.pdf]

Electronic Supplementary Material (ESI) for Chemical Science.
This journal is © The Royal Society of Chemistry 2016

Supplementary Information for:

Mechanism-based inhibition of an aldolase at high concentrations of its natural substrate acetaldehyde: Structural insights and protective strategies

Markus Dick,[a] Rudolf Hartmann,[b] Oliver H. Weiergräber,[b] Carolin Bisterfeld,[a] Thomas Classen,[c] Melanie Schwarten,[b] Philipp Neudecker,[b,d] Dieter Willbold,[b,d] and Jörg Pietruszka[a,c]*

[a]*Institute of Bioorganic Chemistry, Heinrich-Heine-Universität Düsseldorf im Forschungszentrum Jülich, 52425 Jülich, Germany.*
[b]*Institute of Complex Systems, ICS-6: Structural Biochemistry, Forschungszentrum Jülich GmbH, 52425 Jülich, Germany.*
[c]*Institute of Bio- and Geosciences, IBG-1: Biotechnology, Forschungszentrum Jülich GmbH, 52425 Jülich, Germany.*
[d]*Institut für Physikalische Biologie, Heinrich-Heine-Universität Düsseldorf, 40225 Düsseldorf, Germany.*

Contents:

1. SI Methods
2. SI Figures S1-S12
3. SI Tables S1
4. References

1. SI Methods

NMR assignment of reaction products after incubation of DERA with ^{13}C-acetaldehyde.

The integral values are related to crotonaldehyde that has the lowest integral and thus was set to one. The absolute values were determined by integration of aldehyde/olefinic protons.

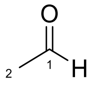

(1)

$\int = 27$

^{1}H NMR (600 MHz, H_2O): δ (ppm) = 2.24 (d, $^3J_{1,2}$ = 3.0 Hz, 3 H, 2′-H), 9.68 (q, $^3J_{1,2}$ = 3.0 Hz, 1 H, 1′-H)

^{13}C NMR (151 MHz, H_2O): δ (ppm) = 32.85 (C-2), 209.49 (C-1)

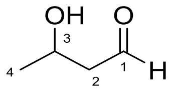

(2)

$\int = 2.3$

^{1}H NMR (600 MHz, H_2O): δ (ppm) = 1.26 (d, $^3J_{4,3}$ = 6.4 Hz, 3 H, 4′-H), 2.66 (ddd, $^2J_{2a,2b}$ = 16.9 Hz, $^3J_{2a,3}$ = 7.8 Hz, $^3J_{2a,1}$ = 2.7 Hz, 1 H, 2′-H_a), 2.71 (ddd, $^2J_{2b,2b}$ = 16.9 Hz, $^3J_{2b,3}$ = 4.6, $^3J_{2b,1}$ = 1.7 Hz, 1 H, 2′-H_b), 4.41 (m*, 1 H, 3′-H), 9.71 (dd, $^3J_{1,2a}$ = 2.7 Hz, $^3J_{1,2b}$ = 1.7 Hz, 1 H, 1′-H)

^{13}C NMR (151 MHz, H_2O): δ (ppm) = 24.89 (C-4), 54.20 (C-2), 65.64 (C-3), 209.43 (C-1)

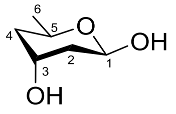

(3a)

$\int = 33$

^{1}H NMR (600 MHz, H_2O): δ (ppm) = 1.21 (d, $^3J_{6,5}$ = 6.3 Hz, 3 H, 6′-H), 1.46 (ddd, $^2J_{4ax,4eq}$ = 14.5 Hz, $^3J_{4ax,5}$ = 11.7 Hz, $^3J_{4ax,3}$ = 3.0 Hz, 1 H, 4′-H_{ax}), 1.54 (ddd, $^2J_{2ax,2eq}$ = 13.6 Hz, $^3J_{2ax,1}$ = 10.2 Hz, $^3J_{2ax,3}$ = 3.0 Hz, 2 H, 2′-H_{ax}), 1.68 (dddd, $^2J_{4eq,4ax}$ = 14.5 Hz, $^3J_{4eq,3}$ = 3.0 Hz, $^3J_{4eq,5}$ = 2.2 Hz, $^4J_{4eq,2eq}$ = 2.2 Hz, 1 H, 4′-H_{eq}), 1.93 (dddd, $^2J_{2eq,2ax}$ = 13.6 Hz, $^3J_{2eq,3}$ = 3.0 Hz, $^3J_{2eq,1}$ = 2.2 Hz, $^4J_{2eq,4eq}$ = 2.2 Hz, 1 H, 2′-H_{eq}), 4.05 (dqd, $^3J_{5,4ax}$ = 11.7 Hz, $^3J_{5,6}$ = 6.3 Hz, $^3J_{5,4eq}$ = 2.2 Hz, 1 H, 5′-H), 4.31 (q, $^3J_{3,2+4}$ = 3.0 Hz, 1 H, 3′-H), 5.10 (dd, $^3J_{1,2ax}$ = 10.2 Hz, $^3J_{1,2eq}$ = 2.2 Hz, 1 H, 1′-H)

^{13}C NMR (151 MHz, H_2O): δ (ppm) = 23.12 (C-6), 40.73 (C-4), 40.82 (C-2), 67.72 (C-3), 70.12 (C-5), 94.58 (C-1)

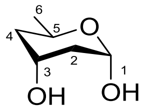

(3b)

$\int = 6.6$

^{1}H NMR (600 MHz, H_2O): δ (ppm) = 1.20 (d, $^3J_{6,5}$ = 6.4 Hz, 3 H, 6′-H), 1.64 (ddd, $^2J_{4ax,4eq}$ = 13.6 Hz, $^3J_{4ax,5}$ = 10.1 Hz, $^3J_{4ax,3}$ = 3.3 Hz, 1 H, 4′-H_{ax}), 1.78 (m, $^2J_{4eq,4ax}$ = 13.6 Hz, $^3J_{4eq,3}$ = 4.0 Hz, $^3J_{4eq,5}$ = 3.0 Hz, 1 H, 4′-H_{eq}), 1.78 (m*, $^2J_{2ax,2eq}$ = X* Hz, $^3J_{2ax,1}$ = X* Hz, $^3J_{2ax,3}$ = 3.0 Hz, 1 H, 2′-H_{ax}), 1.93 (dddd, $^2J_{2eq,2ax}$ = X* Hz, $^3J_{2eq,3}$ = 3.0 Hz, $^3J_{2eq,1}$ = X* Hz, $^4J_{2eq,4eq}$ = X* Hz, 1 H, 2′-H_{eq}), 4.40 (dqd, $^3J_{5,4ax}$ = 10.1 Hz, $^3J_{5,6}$ = 6.4 Hz, $^3J_{5,4eq}$ = 3.0 Hz, 1 H, 5′-H), 4.21 (qui, $^3J_{3,2+4}$ = 4.0 Hz, 1 H, 3′-H), 5.25 (dd, $^3J_{1,2}$ = 3.4 Hz, 1 H, 1′-H_{eq})

^{13}C NMR (151 MHz, H_2O): δ (ppm) = 23.12 (C-6), 38.32 (C-2), 40.70 (C-4), 64.23 (C-5), 66.34 (C-3), 94.17 (C-1)

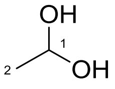

$\int = 45$

(4)

^{1}H NMR (600 MHz, H_2O): δ (ppm) = 1.33 (d, $^3J_{2,1}$ = 5.2 Hz, 3 H, 2’-H), 5.25 (q, $^3J_{2,1}$ = 5.2 Hz, 1 H, 1’-H)

^{13}C NMR (151 MHz, H_2O): δ (ppm) = 25.92 (C-2), 91.00 (C-1)

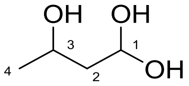

$\int = 3.3$

(5)

^{1}H NMR (600 MHz, H_2O): δ (ppm) = 1.21 (d, $^3J_{4,3}$ = 6.3 Hz, 3 H, 4’-H), 1.73 (m*, 1 H, 2’-H), 1.79 (m*, 1 H, 2’-H), 3.39 (m*, 1 H, 3’-H), 5.18 (dd, $^3J_{1,2a}$ = 6.6 Hz, $^3J_{1,2b}$ = 5.0 Hz, 1 H, 1’-H)

^{13}C NMR (151 MHz, H_2O): δ (ppm) = 25.00 (C-4), 48.37 (C-2), 67.40 (C-3), 91.76 (C-1)

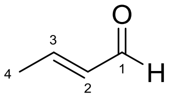

$\int = 1$

(6)

^{1}H NMR (600 MHz, H_2O): δ (ppm) = 2.05 (dd, $^3J_{4,3}$ = 6.8 Hz, $^4J_{4,2}$ = 1.6 Hz, 3 H, 4’-H), 6.22 (ddq, $^3J_{2,3}$ = 15.4 Hz, $^3J_{2,1}$ = 8.3 Hz, $^4J_{2,4}$ = 1.6 Hz, 1 H, 2’-H), 7.20 (dq, $^3J_{3,2}$ = 15.4 Hz, $^3J_{3,4}$ = 6.8 Hz, 1 H, 3’-H), 9.37 (d, $^3J_{1,2}$ = 8.3 Hz, 1 H, 1’-H)

^{13}C NMR (151 MHz, H_2O): δ (ppm) = 21.15 (C-4), 135.8 (C-2), 162.9 (C-3), 201.9 (C-1)

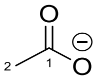

$\int = ?^*$

(7)

^{1}H NMR (600 MHz, H_2O): δ (ppm) = 1.91 (s, 3 H, 2’-H)

^{13}C NMR (151 MHz, H_2O): δ (ppm) = 25.95 (C-2), 184.08 (C-1)

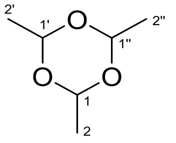

$\int = 90$

(8)

^{1}H NMR (600 MHz, H_2O): δ (ppm) = 1.37 (d, $^3J_{2,1}$ = 5.2 Hz, 3 H, 2’-H), 5.27 (q, $^3J_{1,2}$ = 5.2 Hz, 1 H, 1’-H)

^{13}C NMR (151 MHz, H_2O): δ (ppm) = 22.20 (C-2), 101.70 (C-1)

*Coupling constant or integral could not be measured due to signal overlap.

2. SI Figures

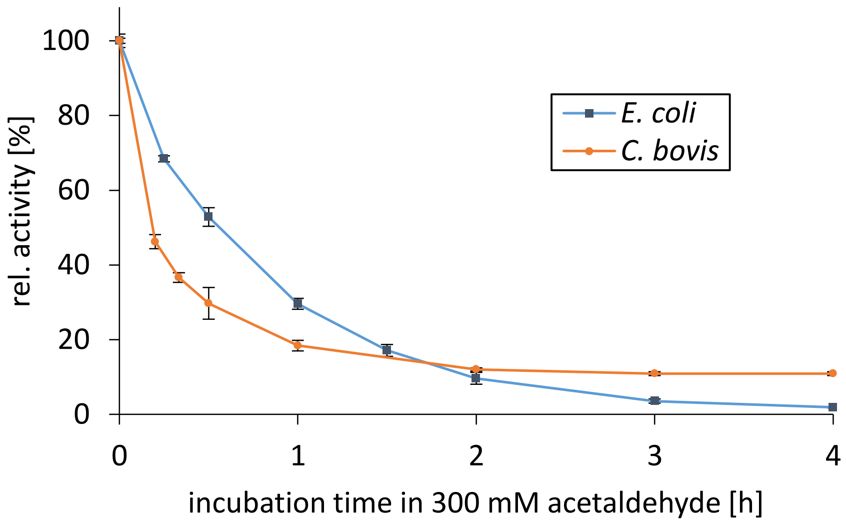

Figure S1. Acetaldehyde tolerance of DERAs from *E. coli* (16 outer lysines) and *C. bovis* (1 outer lysine). The result demonstrates that a lower number of exposed lysine residues does not impart enhanced acetaldehyde stability.

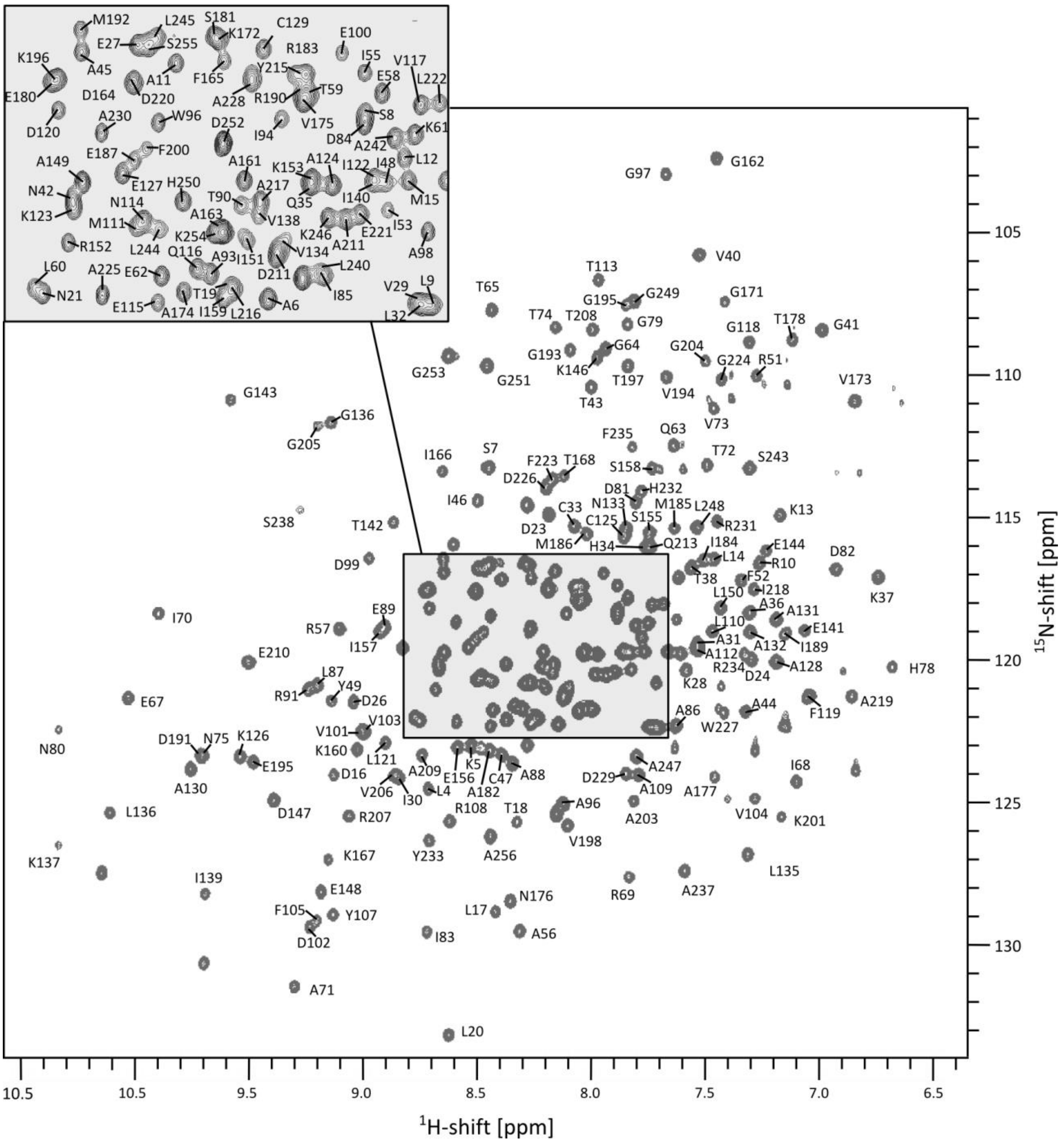

Figure S2. [^{1}H,^{15}N]-TROSY spectrum of [*U*-^{15}N] monomeric *E. coli* DERA (K58E-Y96W). Based on the backbone assignment, signals of the NH-shifts are labelled by their corresponding amino acids.

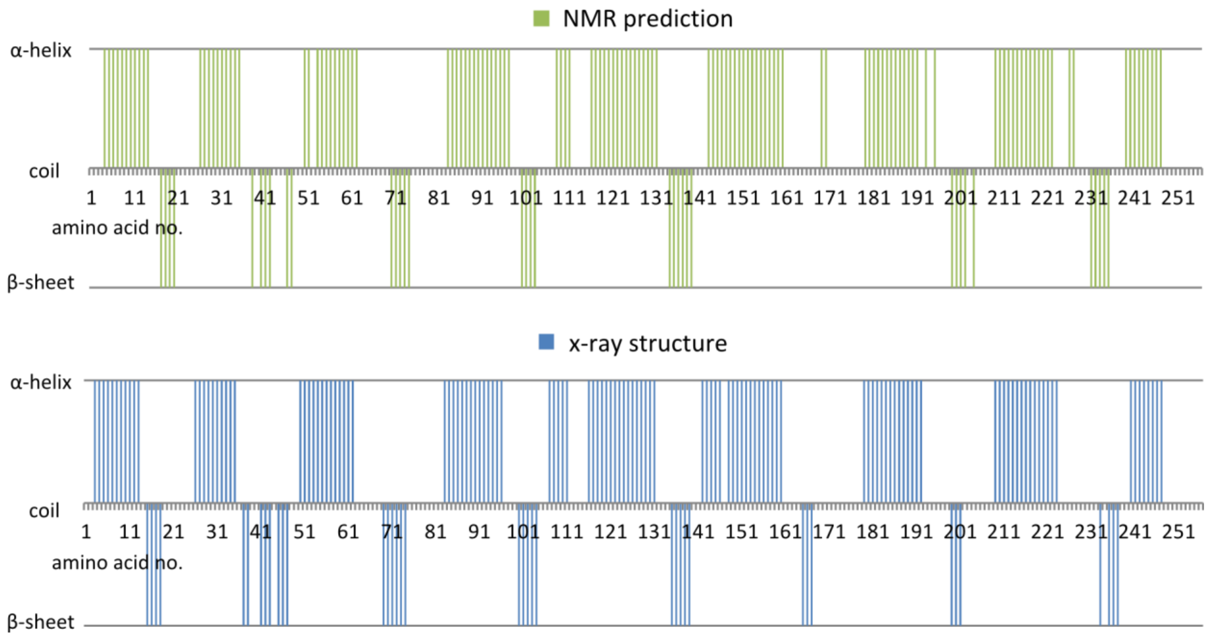

Figure S3. Comparison of the secondary structure prediction by CSI 2.0 and values obtained from the 3D structure of monomeric DERA by DSSP (hydrogen bonding criteria). Both diagrams show a very similar pattern, indicating a correct backbone resonance assignment.

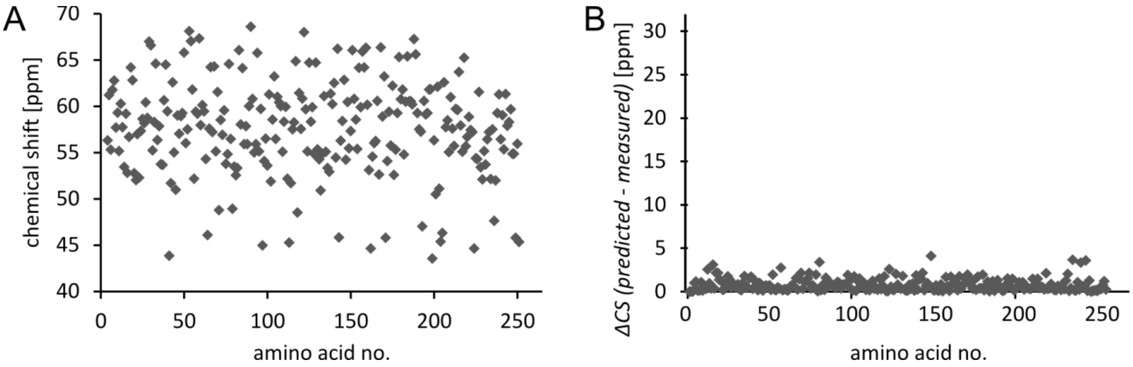

Figure S4. Evaluation of the chemical shifts of C_α atoms derived from HNCA experiments with monomeric DERA. (A) C_α chemical shift of each amino acid assigned by NMR. (B) Chemical shift differences (ΔCS) between the calculated values (predicted by SPARTA based on the 3D structure) and the measured values. Low ΔCS (< 5 ppm for all amino acids) support the correctness of the protein backbone assignment.

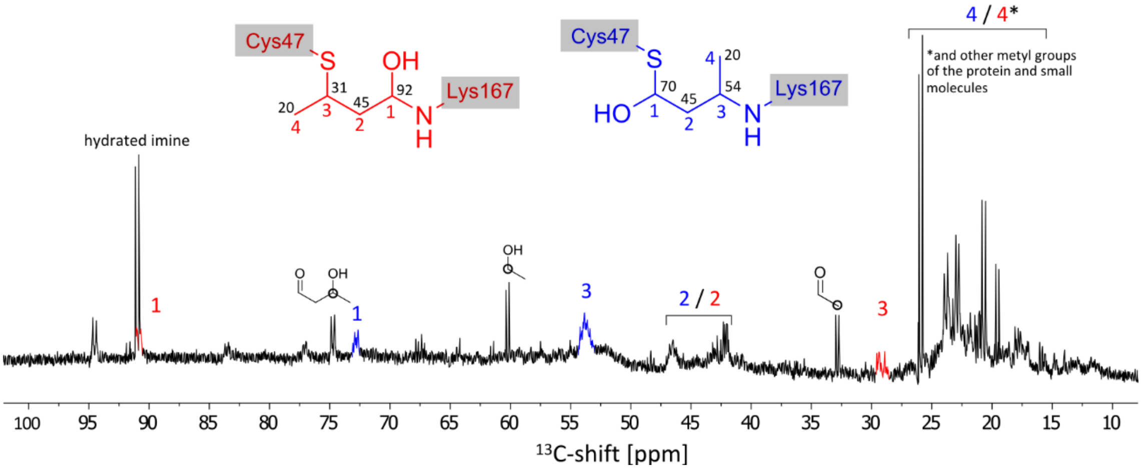

Figure S5. ^{13}C spectrum of unlabelled DERA after incubation with [*U*-^{13}C]-acetaldehyde and removal of free reaction products. The assignment is based on a prediction of chemical shifts.[1] Predicted shifts (in ppm) are indicated in black within the chemical structures of the postulated (red) and the alternative (blue) covalently bound ligand, whereas coloured labels in the spectrum refer to the carbon atoms giving rise to the respective peaks. Sharp signals are probably caused by fast tumbling small molecules while broad peaks can be related to ^{13}C-labeled compounds bound to macromolecules.

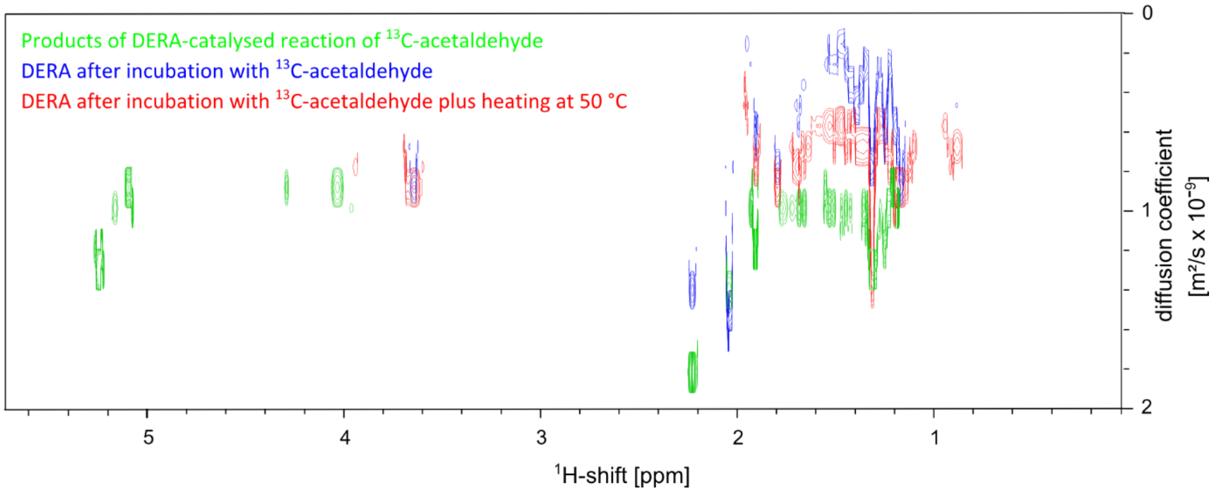

Figure S6. Diffusion-ordered spectroscopy (iDOSY-ctHSQC)[2] of ^{13}C-acetaldehyde incubated DERA before and after heating at 50 °C. Macromolecules such as proteins typically show diffusion coefficients on the order of 10^{-10} m²/s in aqueous solutions, whereas small-molecule species like acetaldehyde and the separated reaction products diffuse at least one order of magnitude faster (green signals).[3, 4] Hence, the blue signals in the iDOSY-ctHSQC spectrum correspond to ^{13}C-labeled compounds which are still bound to the protein after a washing step. While signals at 3.6 ppm are probably caused by remaining glycerol residues in solution, the region of 2-1.8 ppm could be assigned to aliphatic carbon atoms and below 1.5 ppm to methyl groups. After heating at 50 °C, signals between 2-1.8 ppm (red) shift to higher diffusion coefficients, which indicates that the compounds have been (partly) cleaved from the protein.

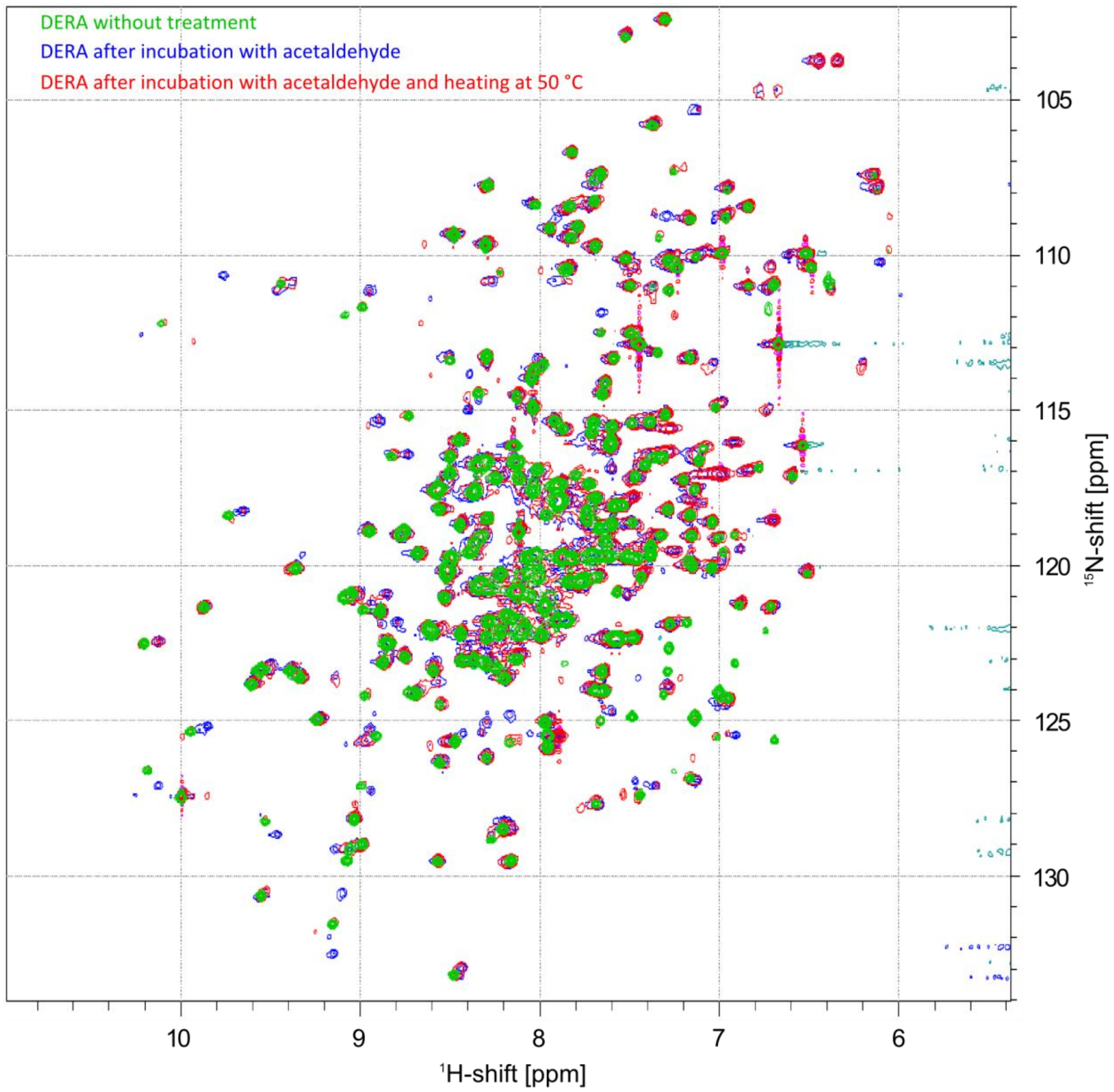

Figure S7. HSQC spectra of [*U*-^{15}N] monomeric *E. coli* DERA (K58E-Y96W) at three different states (untreated; following incubation with acetaldehyde; after an additional heating step).

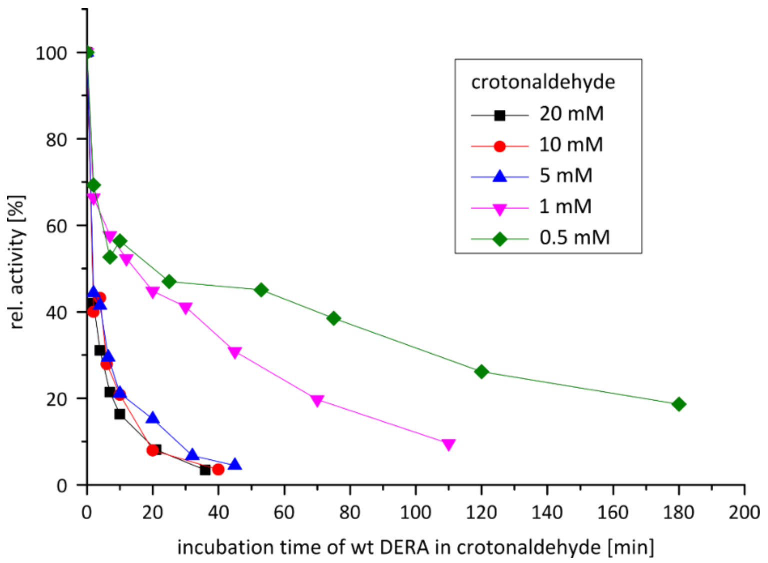

Figure S8. Time-dependent relative activity of wt DERA during incubation with crotonaldehyde at different concentrations.

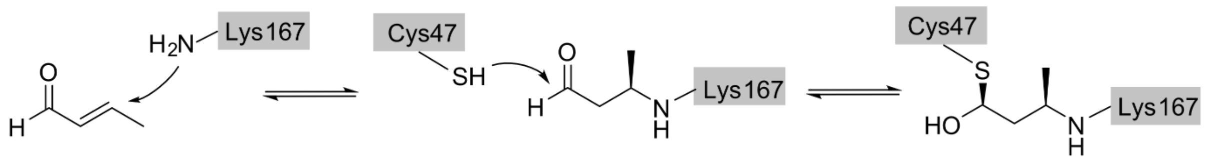

Figure S9. Alternative reaction mechanism leading to DERA inactivation. The nitrogen atom of K167 acts as a nucleophile for the Michael addition to the sp² hybridized C_β atom of crotonaldehyde, while the aldehyde function of crotonaldehyde forms a hemithioacetal with the cysteine residue.

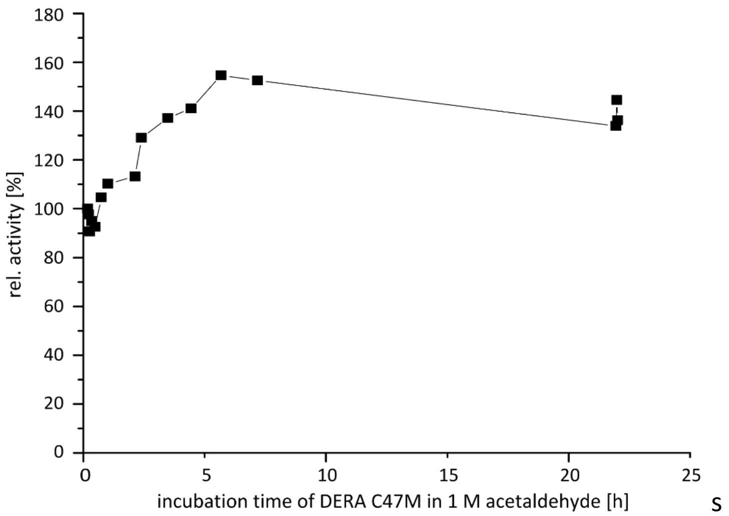

Figure S10. Time-dependent relative activity of DERA C47M during incubation with 1 M acetaldehyde.

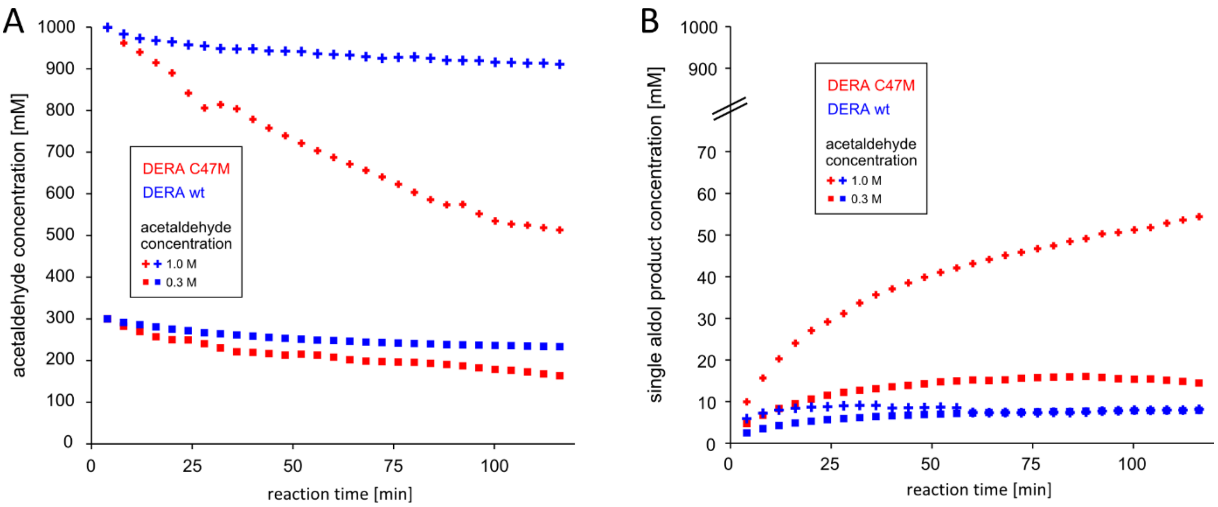

Figure S11. Time dependent (A) acetaldehyde conversion and (B) formation of single aldol product by DERA wt and C47M mutant at different substrate concentrations. Substrate and product concentrations were determined via integration of ^{1}H-signals in the NMR spectra with known initial substrate concentration.

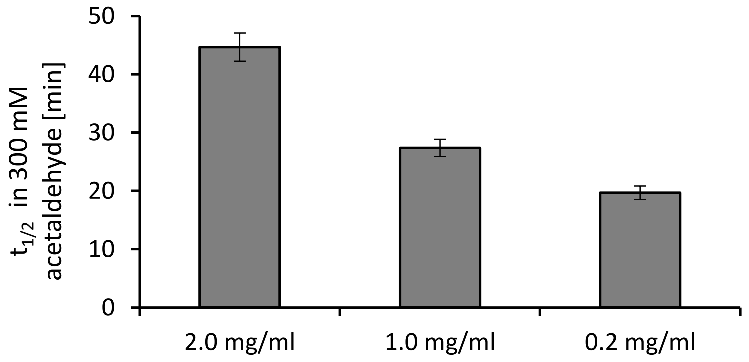

Figure S12. Concentration dependence of acetaldehyde stability of DERA from *E. coli*. The time dependent relative activity was measured during incubation of different DERA concentrations in 300 mM of acetaldehyde. Afterwards the half-life activity with standard deviation was determined as an exponential decay function first order.

3. SI Table

Table S1. X-ray data collection and refinement statistics (monomeric DERA, K58E-Y96W)

	Native	After acetaldehyde incubation	After acetaldehyde incubation and heating
**Data collection**			
Space group	C2	C2	C2
Cell dimensions			
a, *b*, *c* (Å)	110.79, 53.36, 37.84	110.83, 53.30, 37.81	110.78, 53.30, 37.86
β (°)	98.30	98.29	98.31
Resolution (Å)	47.98-1.10 (1.13-1.10)[a]	47.94-1.25 (1.28-1.25)	47.93-1.50 (1.54-1.50)
$CC_{1/2}$ (%)	99.7 (77.5)	99.3 (89.0)	99.6 (62.6)
R_{meas} (%)	6.4 (49.4)	8.8 (38.8)	9.6 (69.1)
$I/\sigma I$	9.78 (2.17)	8.63 (2.68)	8.66 (1.77)
Completeness (%)	97.4 (95.0)	97.7 (90.7)	98.5 (96.3)
Redundancy	2.7	2.9	3.0
**Refinement**			
Resolution (Å)	47.98-1.10	47.94-1.25	47.93-1.50
No. reflections	86,043	58,906	34,541
R_{work} / R_{free} (%)	12.11 / 14.36	11.66 / 14.37	15.03 / 17.48
No. atoms[b]			
Protein	2118	2081	2051
Ligands	27	20	20
Water	353	338	306
B-factors (Å^2)			
Protein	13.0	14.0	17.1
Ligands	12.8	17.0	23.5
Water	30.0	31.8	32.1
R.m.s. deviations			
Bond lengths (Å)	0.010	0.010	0.007
Bond angles (°)	1.187	1.176	0.887

[a] Values in parentheses are for highest-resolution shell.

[b] Numbers include alternative conformers.

4. References

1. B. Shoulders and S. C. Welch, *J. Chem. Educ.*, 1987, **64**, 915-918.
2. A. S. McLachlan, J. J. Richards, A. R. Bilia and G. A. Morris, *Magn. Reson. Chem.*, 2009, **47**, 1081-1085.
3. T. Brand, E. J. Cabrita and S. Berger, *Theory and Application of NMR Diffusion Studies*, Springer, 2006.
4. V. V. Krishnan, *J. Magn. Reson.*, 1997, **124**, 468-473.
